# How to individualize device therapy in transthyretin cardiac amyloidosis: A dyssynchrony-based case report

**DOI:** 10.1016/j.hrcr.2026.07.015

**Published:** 2026-07-29

**Authors:** Ahmet Taha Sahin, Ahmet Lutfu Sertdemir, Yakup Alsancak, Mustafa Celik, Enes Elvin Gul

**Affiliations:** Department of Cardiology, Necmettin Erbakan University, School of Medicine, Konya, Türkiye

**Keywords:** Cardiac amyloidosis, Cardiac physiologic pacing, Device, Dyssynchrony, Ultra-high-frequency electrocardiogram

**Key Teaching Points**

• Significant ventricular electrical dyssynchrony may be present despite only mildly prolonged QRS duration on standard 12-lead electrocardiogram (ECG).
• Ultra-high-frequency ECG-based ventricular dyssynchrony imaging may provide incremental information beyond conventional QRS duration assessment and help characterize the ventricular dyssynchrony substrate.
• Device therapy in transthyretin amyloid cardiomyopathy should be individualized. Advanced dyssynchrony assessment may assist in selecting cardiac physiologic pacing strategies in selected patients, although further studies are required before routine adoption.

## Introduction

Cardiac amyloidosis is frequently complicated by conduction system disease, ventricular arrhythmias, and progressive heart failure. However, selecting the optimal device strategy remains challenging, particularly in patients with only mildly prolonged QRS duration. This case highlights how advanced ventricular dyssynchrony assessment using ultra-high-frequency electrocardiogram (ECG) influenced device selection and guided an individualized cardiac physiologic pacing strategy in a patient with transthyretin cardiac amyloidosis.

## Case presentation

A 42-year-old woman with a history of non-ischemic dilated cardiomyopathy, transthyretin cardiac amyloidosis (diagnosed in October 2025), permanent atrial fibrillation, and prior transient ischemic attack without residual deficit (2023) was referred for device therapy evaluation due to advanced heart failure symptoms. Subsequent ambulatory rhythm monitoring demonstrated frequent non-sustained ventricular tachycardia episodes.

The patient had New York Heart Association (NYHA) functional class III symptoms despite guideline-directed medical therapy including oral Tafamidis 61 once daily. Transthoracic echocardiography demonstrated severe left ventricular systolic dysfunction with a left ventricular ejection fraction of 20%. Baseline ECG revealed atrial fibrillation with nonspecific intraventricular conduction delay and a QRS duration of 127 ms. Laboratory testing was unremarkable except for a markedly elevated N-terminal pro–B-type natriuretic peptide level of 6600 pg/mL. Pre-procedural ventricular dyssynchrony imaging (VDI) demonstrated significant ventricular electrical dyssynchrony despite only mildly prolonged QRS duration on the 12-lead ECG (Figure 1).

**Figure 1:**
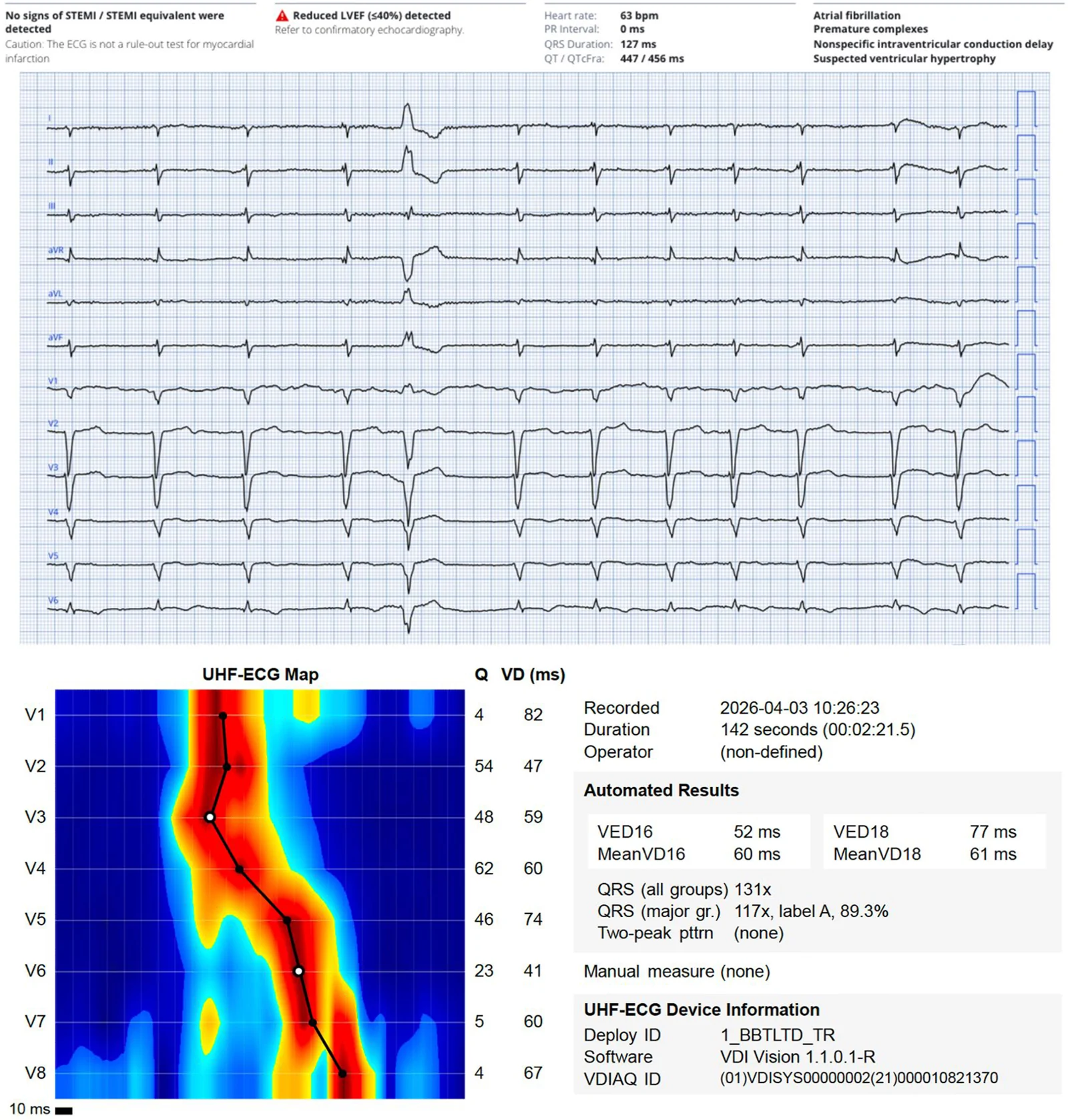
Baseline electrocardiographic and ventricular dyssynchrony assessment in a patient with transthyretin cardiac amyloidosis. *Top*: 12-lead electrocardiogram demonstrating atrial fibrillation with mildly prolonged QRS duration (127 ms); ECG signal enhancement and visualization were performed using PMcardio software. *Bottom*: Ventricular dyssynchrony imaging analysis demonstrating significant interventricular (VED18: 77 ms) electrical dyssynchrony despite only modest QRS prolongation.

What would be your preferred device strategy in this patient with transthyretin cardiac amyloidosis (ATTR-CM), severe left ventricular dysfunction, ventricular arrhythmias, and a baseline QRS duration of 127 ms?

A. Single-chamber implantable cardioverter-defibrillator (ICD) implantation for primary prevention
B. Subcutaneous ICD (S-ICD) implantation
C. Cardiac physiologic pacing
D. Conservative follow-up without device implantation

## Case discussion

Cardiac amyloidosis is increasingly recognized as a complex infiltrative cardiomyopathy characterized not only by progressive myocardial dysfunction but also by substantial involvement of the cardiac conduction system.^1^^,^^2^ Amyloid infiltration may lead to atrioventricular conduction delay, bundle branch block, atrial fibrillation, ventricular arrhythmias, and progressive electromechanical dyssynchrony.^3^

Previous studies have demonstrated that electrical dyssynchrony is relatively common in cardiac amyloidosis and is associated with worse clinical outcomes, including advanced heart failure symptoms, increased pacing requirements, and higher mortality. Martens et al^4^ evaluated more than 1100 patients with cardiac amyloidosis and demonstrated that approximately 20% exhibited significant electrical dyssynchrony, most commonly in the form of bundle branch block or intraventricular conduction delay. Importantly, the presence of dyssynchrony was associated with worse NYHA functional class and adverse clinical outcomes. Furthermore, patients with ATTR-CM demonstrated a progressively higher prevalence of dyssynchrony with advancing disease stage, emphasizing the close relationship between infiltrative myocardial remodeling and conduction system disease.

Cardiac physiologic pacing is defined as any form of cardiac pacing intended to restore or preserve ventricular synchrony either with conduction system pacing (CSP) or cardiac resynchronization therapy (CRT).^5^ In recent years, CSP, particularly left bundle branch area pacing (LBBAP), has emerged as an attractive alternative in cardiac amyloidosis because of its ability to preserve physiological ventricular activation. Pham-Trung et al^6^ demonstrated that LBBAP is feasible and safe in cardiac amyloidosis, achieving significant QRS narrowing with stable pacing parameters during follow-up.^6^ Similarly, recent contemporary data from the LEFT-RIGHT CA study suggested that LBBAP may reduce heart failure worsening and acute decompensated heart failure events compared with conventional right ventricular pacing, despite a higher ventricular pacing burden.^7^ A recent review further emphasized that LBBAP may help preserve ventricular synchrony and potentially stabilize heart failure progression in patients with ATTR-CM with conduction disease.^8^

An important aspect of the present case is the use of advanced dyssynchrony assessment with the help of ultra-high-frequency ECG (UHF-ECG) beyond conventional QRS duration analysis. Although surface ECG parameters, such as QRS duration and morphology, are routinely used for patient selection, they provide only limited insight into the complex intraventricular activation patterns. UHF-ECG provides superior temporal resolution of ventricular activation and allows precise assessment of electrical dyssynchrony beyond standard QRS duration.^9^ Baseline VDI demonstrated marked interventricular dyssynchrony (VED18 77 ms) despite a baseline QRS duration of 127 ms. Following CRT-D implantation, the paced QRS duration was 136 ms, whereas post-procedural VDI demonstrated improved interventricular synchrony with VED18 decreasing to −29 ms. Therefore, UHF-ECG may provide incremental information regarding ventricular activation patterns and aid in the characterization of ventricular dyssynchrony beyond conventional QRS duration assessment. However, these findings should be interpreted cautiously. In the EchoCRT trial, patients with heart failure, left ventricular ejection fraction of ≤35%, and QRS duration of <130 ms, and echocardiographic dyssynchrony did not derive clinical benefit from CRT. Therefore, our observation should be considered hypothesis-generating only.^10^ In the present case, despite a baseline QRS duration of only 127 ms, advanced ventricular dyssynchrony was identified through detailed VDI assessment, ultimately influencing device selection toward cardiac physiologic pacing rather than conventional ICD therapy. This case highlights the potential dissociation between conventional surface ECG findings and the underlying mechanical–electrical substrate.

In the present case, the combination of severe left ventricular dysfunction, recurrent ventricular tachyarrhythmias, and significant dyssynchrony identified on VDI supported a CRT-D strategy rather than isolated ICD implantation. An initial attempt at left bundle branch area pacing using a lumenless lead (Medtronic 3830, Medtronic, MN) was unsuccessful despite evaluation of multiple septal positions, likely reflecting extensive septal infiltration and conduction system involvement. Consequently, conventional biventricular CRT-D implantation was successfully performed with an optimal lateral coronary sinus position (Figure 2A and 2B). Post-procedure VDI showed significant improvement of interventricular dyssynchrony (Figure 3). The patient had been receiving tafamidis for approximately 6 months before device implantation and was maintained on maximally tolerated guideline-directed medical therapy. No major changes in heart failure medical therapy, including diuretic dosing, were made immediately before implantation or during the first month after implantation. At 1-month follow-up, NYHA functional class improved from III to II and N-terminal pro–B-type natriuretic peptide decreased from 6600 pg/mL to 3100 pg/mL. However, given the single-case design and short follow-up, these findings should be interpreted cautiously and cannot be attributed solely to CRT-D implantation.

**Figure 2:**
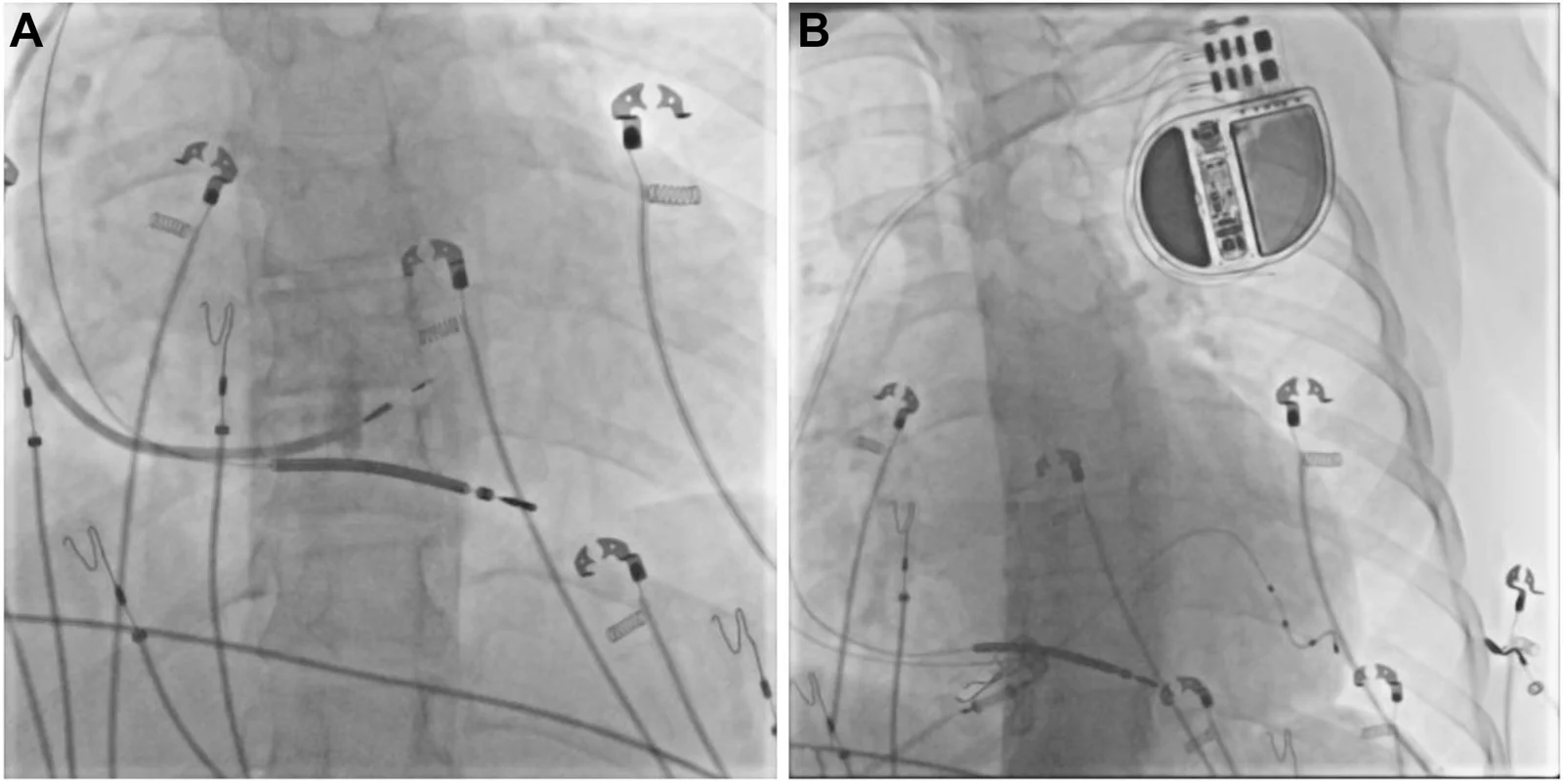
Fluoroscopic images demonstrating implantation of left bundle branch area pacing (LBBAP) and coronary sinus leads. **A:** Septogram revealing deep penetration of LBBAP lead (Medtronic 3830, Medtronic, MN). **B:** Final fluoroscopic image after successful cardiac resynchronization therapy implantation showing stable right ventricular shock lead and quadripolar left ventricular lead in the lateral branch.

**Figure 3:**
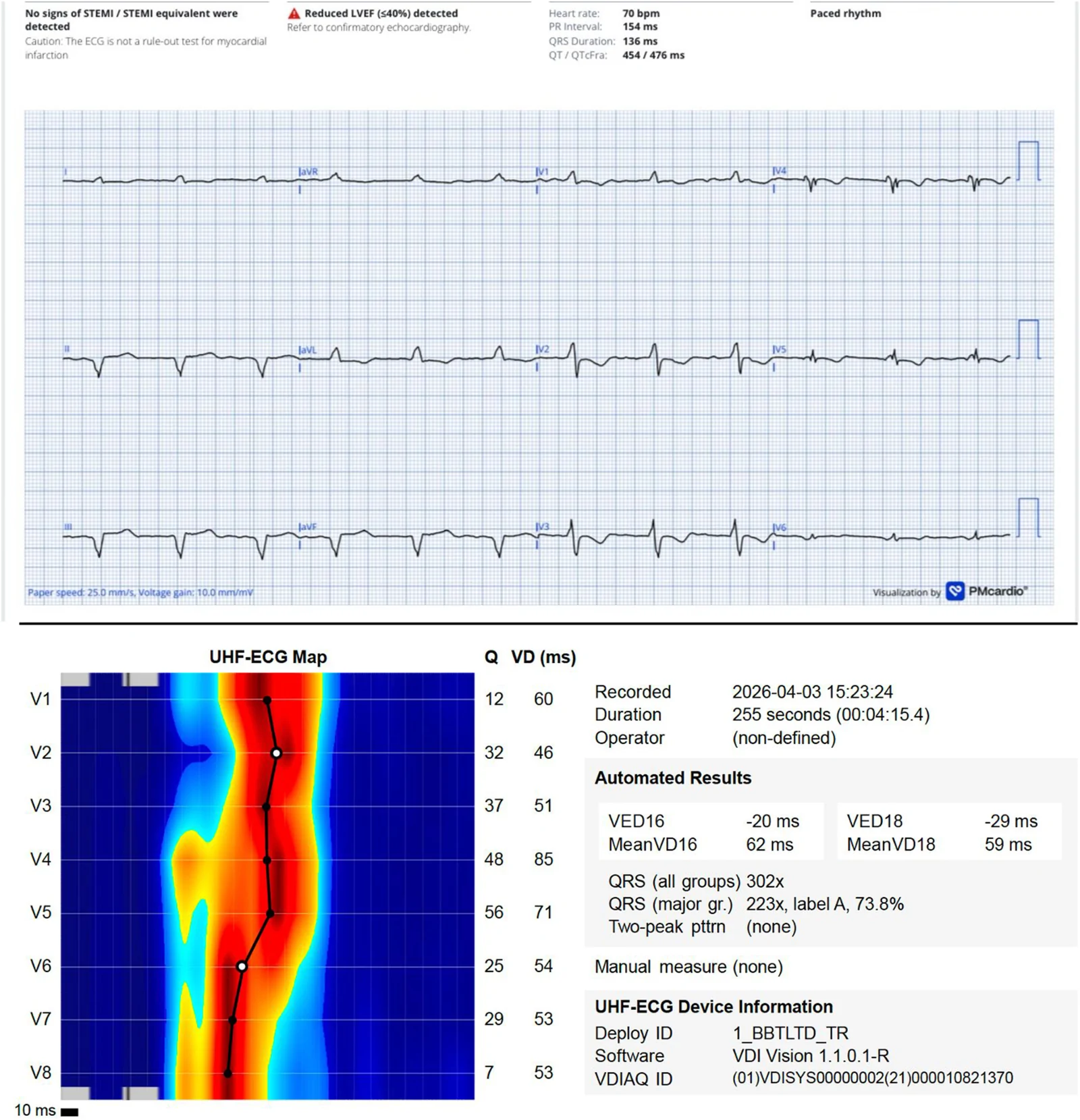
Post-procedural electrocardiographic and ventricular synchrony assessment following cardiac resynchronization therapy with defibrillator (CRT-D) implantation. *Top*: Post-implantation 12-lead electrocardiogram demonstrating biventricular paced rhythm with paced QRS duration of 136 ms; electrocardiogram signal enhancement and visualization were performed using PMcardio software. *Bottom*: Post-procedural ventricular dyssynchrony imaging analysis demonstrating improved interventricular dyssynchrony and ventricular electrical synchrony after CRT-D implantation (VED18: −29 ms). CRT-D = cardiac resynchronization therapy-defibrillator; VDI = ventricular dyssynchrony imaging.

## Conclusion

Cardiac amyloidosis may be associated with significant ventricular dyssynchrony even in the absence of markedly prolonged QRS duration. Therefore, advanced dyssynchrony assessment and individualized resynchronization strategies may play an important role in device selection and heart failure management in patients with advanced ATTR-CM.

## Disclosures

The authors declare no conflict of interest.
